# Seeking insights into the EPidemiology, treatment and Outcome of Childhood Arthritis through a multinational collaborative effort: Introduction of the EPOCA study

**DOI:** 10.1186/1546-0096-10-39

**Published:** 2012-11-20

**Authors:** Alessandro Consolaro, Nicolino Ruperto, Giovanni Filocamo, Stefano Lanni, Giulia Bracciolini, Marco Garrone, Silvia Scala, Luca Villa, Giuseppe Silvestri, Daniela Tani, Alessandra Zolesi, Alberto Martini, Angelo Ravelli

**Affiliations:** 1 Istituto Giannina Gaslini, Genova, Italy; 2 Policlinico S. Orsola-Malpighi, Bologna, Italy; 3 Università degli Studi di Genova, Genova, Italy

**Keywords:** Juvenile idiopathic arthritis, Epidemiology, Treatment, Outcome

## Abstract

The epidemiology of juvenile idiopathic arthritis (JIA) is variable worldwide. In particular, a wide disparity exists in the prevalence of the diverse disease subtypes across different geographic areas. The therapeutic approach to JIA is not standardized and no established and widely accepted guidelines are available. In the past decade, there have been important progresses in the management of the disease, but the availability of the novel and costly biologic medications is not uniform throughout the world. This issue may have significant impact on disease prognosis, with children living in poorer countries being at greater risk of accumulating disease- and treatment-related damage than children followed in Western pediatric rheumatology centers. The multinational study of the EPidemiology, treatment and Outcome of Childhood Arthritis (EPOCA study) is aimed to obtain information on the frequency of JIA subtypes in different geographic areas, the therapeutic approaches adopted by pediatric rheumatologists practicing in diverse countries or continents, and the disease and health status of children with JIA currently followed worldwide. Parent- and child-reported outcomes are meant to be recorded through the administration of a new multidimensional questionnaire, the Juvenile Arthritis Multidimensional Assessment Report (JAMAR). The first step of the study is based on the translation and cross-cultural adaptation of the questionnaire in the national language of each participating country. Each center is, then, asked to enroll a sample of consecutive JIA patients, who should undergo a retrospective assessment and a cross-sectional evaluation, including completion of the JAMAR, a standardized joint examination, and the assessment of articular and extra-articular damage. At the end of May 2012, 124 centers in 55 countries have agreed to participate in the study. The JAMAR has been or is currently being translated in 38 national languages. The target patient sample is more than 10,000 JIA children worldwide.

## Review

### Background

Juvenile idiopathic arthritis (JIA) is a chronic inflammatory disease of presumed autoimmune etiology that affects ~1 of 1,000 children worldwide [1]. It is the most common chronic rheumatic disease in the pediatric age and an important cause of short-term and long-term disability. It is a heterogeneous condition, which is classified in 7 different categories based on the clinical manifestations observed in the first 6 months after onset [2,3]. Some of these categories may represent different diseases, characterized by distinct modalities of presentation, clinical features, immune-pathogenesis and, in some cases, genetic background. The current International League of Associations of Rheumatology (ILAR) classification of JIA [2,3] has the merit of having solved the drawback of the previous heterogeneity in nomenclature and criteria between Europe and North America. However, it has been subject to several criticisms. In particular, it has been argued that the clinical parameters used to separate patients in the various categories, namely the number of affected joints and the presence of psoriasis, may not lead to identify homogeneous disease subgroups [4].

The epidemiology of JIA is known to be variable worldwide. In particular, a wide disparity exists in the prevalence of the diverse disease subtypes between different countries. Although in the Western world the most common category is represented by oligoarthritis, this subtype is rare in countries such as Costa Rica, India, New Zealand, and South Africa, where polyarthritis predominates [5-8]. Similar discrepancies have been reported for juvenile spondyloarthropaties and for iridocyclitis, which is the main extra-articular complication of some JIA subsets, particularly those characterized by the presence of antinuclear antibodies (ANA). However, little data exists on the epidemiology of JIA. Furthermore, most studies have involved single countries and the comparison of the distribution of JIA categories across different geographic areas has seldom been attempted.

A number of therapeutic options are available for the management of JIA, ranging from NSAIDs to systemic or intra-articular corticosteroids, to the traditional disease modifying antirheumatic drugs (DMARDs), to the novel biologic agents [1,9-11]. However, although treatment recommendations and standards of care for JIA have recently been published [12,13], the therapeutic approach to JIA is not standardized and no treatment guidelines have been prospectively evaluated, internationally accepted, and widely and systematically implemented. As a result, the therapeutic choices for the management of individual patients are likely variable between pediatric rheumatologists practicing in single or different countries.

In the past decade, there have been several important advances in the management of JIA. In particular, the introduction of biologic agents holds great promise for the treatment of patients who are at greatest risk of developing irreversible structural damage to joints and permanent physical disability. A recent CARRAnet study found that 45% of JIA patients in the US are exposed to biologic DMARDs [14]. However, the availability of biologic drugs is not uniform around the world. Due to their high cost, these medications or some of them may not be afforded by the national health care system of poorer countries or by the families of children living in these countries. As a result, many children with JIA around the world may not entirely benefit from the recent progresses in disease treatment. For some of these children, particularly those with the more severe systemic or polyarticular subtypes, the administration of high and prolonged doses of corticosteroids may be the sole therapeutic option to control disease activity symptoms. As a consequence, they are exposed to a high risk of experiencing serious and potentially devastating corticosteroid-related side effects. Altogether, these issues may have significant impact on disease prognosis and may place children living in poorer countries at greater risk of accumulating disease-related or treatment-related damage than children followed in Western pediatric rheumatology centers.

Owing to the aforementioned differences in epidemiology, treatment approaches, and availability of medications, there is likely an important variability in the outcome of children with JIA around the world. Although a number of long-term outcome surveys have been carried out in the last 4 decades [15-17], most of these studies have involved single centers or a few countries in the same geographic area (e.g., Scandinavia). The comparison of JIA disease outcomes between different parts of the world has never been attempted. Furthermore, most studies have evaluated patients with many years of disease duration, whereas the outcomes achieved with the present treatment, that is, the disease status of children currently followed in pediatric rheumatology centers, has seldom been investigated [18].

An important shortcoming of existing outcome studies lies in the use of a wide variety of clinical measures, which hampers comparability of results [16]. Furthermore, most analyses have focused only on specific aspects of disease outcome, such as remission, functional disability, radiographic damage, or health-related quality of life (HRQL). In addition, children’s self-reported assessments have been seldom incorporated.

Most clinical measures currently used to assess the disease status, particularly functional ability and HRQL questionnaires, are lengthy and complex [19]. For this reason, they are rarely applied in standard clinical practice to assess patient status and to monitor therapeutic response and disease course over time. However, it has been recommended that regular use of quantitative measures in daily practice may lead to improve the quality of patient care. To give an example, it has been suggested that the implementation of a treat-to-target strategy, based on treatment escalation if goal Juvenile Arthritis Disease Activity Score (JADAS) [20,21] is not reached, in clinical practice may improve disease outcome [22]. Furthermore, it is widely agreed that integration of parent- and child-reported outcomes in daily care may facilitate concordance with physician’s choices and compliance with therapeutic prescriptions [23-25]. To reach this goal, there is a need for outcome measures that are simple and easy to apply. In addition, to facilitate comparability of outcome data across different centers, such instruments need to be widely agreed upon and should be translated, cross-culturally adapted and validated in different languages according to established international guidelines [26].

Recently, a new multidimensional questionnaire that includes all main parent- and child-reported outcomes used in the clinical evaluation of children with JIA has been developed [27]. This tool, named Juvenile Arthritis Multidimensional Assessment Report (JAMAR), may be well suited to collect parent- and child-reported information in standard clinical care.

## Aims of the study

To address the aforementioned issues, a multinational study of the EPidemiology, treatment and Outcome of Childhood Arthritis (EPOCA study) has been launched. This project is primarily aimed to obtain information on the frequency of JIA subtypes in different geographic areas, the therapeutic approaches followed by pediatric rheumatologists from diverse countries or continents, and the current disease and health status of children with JIA followed worldwide. Additional objectives are to investigate the availability of biologic medications in different countries, to foster the regular use of standardized quantitative clinical measures in the clinical assessment of children with JIA in standard clinical care, and to promote the embracement of a uniform set of outcome measures across international pediatric rheumatology centers.

## Methods

### Study design and patient selection

To obtain figures generalizable on a worldwide basis, the involvement of a large number of countries is aimed for. To reach this goal, participation in the study has been first proposed to the national coordinating center of all countries belonging to the Pediatric Rheumatology International Trials Organization (PRINTO), and to one pediatric rheumatology center in the US and Canada. Once the contacted center had agreed to participate in the study, the center was asked to offer the participation in the study to any other pediatric rheumatology center in the country that were meant to be potentially interested in joining the project.

To ensure that the data regarding the epidemiology, treatment and outcome of JIA are reliable, the collection of a representative sample of the patients followed at each participating center is planned. Each centre is, then, asked to enroll 100 consecutive and unselected patients meeting the ILAR criteria for JIA or, if the centre does not expect to see at least 100 patients within 6 months, to enroll all consecutive and unselected patients meeting the same criteria seen within the first 6 months after the study start.

### Translation and cross-cultural adaptation of the JAMAR

Because the parent- and child-reported outcomes are meant to be recorded through the administration of the JAMAR, the first step of the study was based on the translation and cross-cultural adaptation of the questionnaire in the national language of each participating country. The translation of the questionnaire is conducted following a standard procedure [26]. In brief, cross-culturally adapted translation in the national language of the country of the standard-English version of the parent and child versions of the JAMAR is first made by at least 2 (preferably 3) independent translators. The forward translators and 1 or 2 other individuals not involved in translation procedures then meet to reach consensus, in order to obtain preliminary unified versions from the forward translations. Next, the preliminary version of the questionnaires is back-translated by at least 2 (preferably 3) independent native-English translators. Backward translations are reviewed by the PRINTO Coordinating Center staff, with specific expertise in translations issues, to verify their correspondence with the original standard-English versions of the questionnaire. The PRINTO investigators then send their comments to the national coordinator. Local forward and backward translators finally meet to discuss and reconcile the comments received from the PRINTO staff. The purpose of the meeting is to reach consensus among translators on the second unified translation of the questionnaires.

In the subsequent step, the second versions of the JAMAR is administered, using a probe technique, to 10 parents of children with JIA and to 10 children of different educational background, to confirm parents’ and children’s understanding of questionnaire items among the target population. A health professional, familiar with the meaning of each item, submits the questionnaire to parents and children and asks them to review each question. The questions that are not correctly interpreted by 20% or more of the parents or children are reviewed by the national coordinating center and revised accordingly. In case modifications to the second unified forward version are needed, all forward and backward translators convene in a final meeting to discuss the results of the probe technique. The purpose of this meeting is to reach consensus among translators on the final unified national language-version of the questionnaires.

### Study assessments

Each JIA patient enrolled in the study should receive a retrospective evaluation, based on the review of the clinical chart, and a cross-sectional assessment, made by the attending physician at the time of the study visit. Prior to the study visit, a parent of the child and the child him/herself, if aged at least 7-8 years, should complete the national-language translation of the parent proxy-report and child self-report version of the JAMAR, respectively. The parents or guardians of all children or the children themselves (as appropriate) should provide written informed consent to participation in the study. Each participating center should have obtained the approval of the study protocol from the local ethics committee if required by the national laws.

Retrospective assessments include: demographic data; ILAR category and detail of ILAR criteria met by the patient, including descriptors/exclusions and history of iridocyclitis; positive family history for autoimmune diseases; results of ANA, rheumatoid factor, and HLA-B27 determinations; history of macrophage activation syndrome (MAS) or other co-morbid conditions; registration of drug therapies received by the patient from disease onset to cross-sectional assessment. Information regarding the availability of specific biologic medications in each country will be sought for through separate survey.

Cross-sectional assessment includes a standardized joint examination, the physician’s global rating of the level of disease activity, the assessment of the presence of ongoing enthesitis, dactylitis, active uveitis, and MAS, the physician’s rating of disease status (remission, flare, continued disease activity) and course (much or slightly improved, stable, slightly or much worsened) on categorical scales, the registration of the therapeutic decisions made by the attending physician at the end of the visit, and the assessment of the amount of articular and extra-articular damage through the Juvenile Arthritis Damage Index (JADI) [28]. Acute phase reactants are tested if clinically indicated.

The JAMAR [27] includes the following 15 measures/items: 1) assessment of physical function through the JAFS [19]; 2) rating of the intensity of child’s pain on a 21-numbered circle VAS [29]; 3) assessment of HRQL, through the PRQL [30]; 4) rating of child’s overall well-being on a 21-numbered circle VAS [29]; 5) assessment of the presence of pain or swelling in the following joints or joint groups: cervical spine, lumbo-sacral spine, shoulders, elbows, wrists, small hand joints, hips, knees, ankles, and small foot joints; 6) assessment of morning stiffness; 7) assessment of extra-articular symptoms (fever and rash); 8) rating of the level of disease activity on a 21-numbered circle VAS [29]; 9) rating of disease status at the time of the visit as remission, continued activity or relapse; 10) rating of disease course from previous visit as much improved; slightly improved; stable; slightly worsened; or much worsened; 11) check-list of the medications the child is taking; 12) check-list of side effects of medications; 13) report of difficulties with medication administration; 14) report of school problems caused by the disease; 15) a question about satisfaction with the outcome of the illness [31]. The JAMAR is available in 2 versions, one for parent proxy-report and one for child self-report, with the suggested age range of 7-18 years for use as self-report.

To compare the parent- and child-reported information obtained for children with JIA with that of a healthy children population, each national coordinating center is asked to administer the JAMAR to around 100 parents of healthy children and around 100 healthy children aged more than 7-8 years. This sample can be represented by the healthy brothers and sisters, other relatives (e.g. cousins), or friends of JIA patients included in the study. In case of healthy brothers and sisters of JIA patients, the parent is asked to complete the JAMAR for both the patient and the healthy sibling(s) at the time of the visit. The healthy sibling(s) can complete the JAMAR at the time of the visit (if present) via tablet, personal computer in the waiting room or at home via internet (a user specific password is automatically generated by the PRINTO web database). Data are collected on an sql web database placed on an https platform on the dedicated PRINTO server. Centres are allowed to collect data via web through the PRINTO member area or alternatively on paper.

## Preliminary results

**Figure 1:**
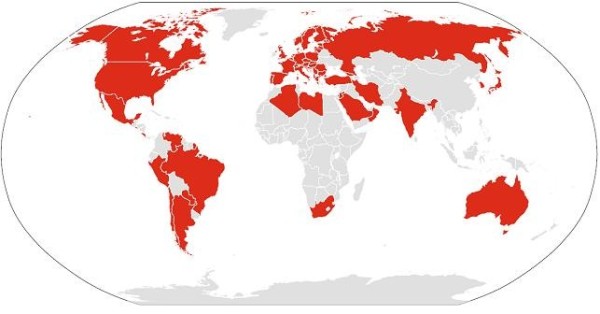
Geographic distribution of the countries involved in the EPOCA study.

At the end of October 2012, 124 centers in 54 countries have shown their interest to participate in the study (Table 1 and Figure 1). The JAMAR has been or is currently being translated and cross-cultural adapted in 38 national languages. The number of completed translations is 33. Data collection has already been started in 18 countries, and has been completed in 4 countries with more than 1,800 JIA patients and nearly 400 healthy controls already collected. The study is planned to be completed by June 2013 and the results of data analyses will likely be available by the end of 2013. The target patient sample is more than 10,000 children worldwide.

**Table 1:** List of the countries involved in the EPOCA study

Albania	Finland	Oman
Algeria	France	Paraguay
Argentina	Georgia	Peru
Australia	Germany	Poland
Austria	Greece	Portugal
Belgium	Hungary	Romania
Brazil	India	Russian Federation
Bulgaria	Iran	Saudi Arabia
Canada	Israel	Serbia
Chile	Italy	Slovakia
Colombia	Japan	Slovenia
Costa Rica	Jordan	South Africa
Croatia	Latvia	Spain
Czech Republic	Libya	Sweden
Denmark	Lithuania	Switzerland
Egypt	Mexico	Turkey
El Salvador	Netherlands	United Kingdom
Estonia	Norway	USA

## Study limitations

So far, only a few pediatric rheumatology centers in the United States and Canada have been included in the study. Therefore, the information collected for this geographic area will be very limited and will not enable the comparison of the therapeutic approaches across different institutions within each country. This limitation will also hamper the appreciation of whether the governmental cost restrictions in Canada might affect the prescription policy regarding biologic medications in Canadian versus US centers. To reduce this shortcoming of the study, we plan to include more North American centers in the next future. We recognize that because most centers involved in the study belong to PRINTO, the study is dependent on the referrals to the PRINTO-related pediatric rheumatologists. Furthermore, the results of the epidemiologic analysis will reflect the epidemiology of patients referred to pediatric rheumatologists and will not account for patients referred to adult rheumatologists and general pediatricians. We should finally acknowledge that although the study is intended to be a global project, it is neither aimed to optimize recruitment in areas where there is not pediatric rheumatologists nor to establish recommendations or standards of care for JIA.

## Conclusions

The EPOCA study is a huge and ambitious effort aimed to get an overview on a worldwide basis of the geographic distribution of the various JIA phenotypes, the treatment approaches adopted by international pediatric rheumatologists, and the current outlook of children with JIA followed in pediatric rheumatology centers in different countries. The achievement of large-scale figures on the frequency of JIA categories throughout the word may provide the rationale for devising changes in the present disease classification and for refining the strategy of future genetic and pathogenetic investigations. The comparison of the management policies of pediatric rheumatologists practicing in diverse geographic and clinical settings may stimulate the need for a better standardization of the therapeutic approaches and the development of data-driven management guidelines. In working out the data, particular attention will be paid to the evaluation of the relationship between the availability of biologic medications in a particular country and the outcome of children followed in the country. The information obtained with the EPOCA study will help make national and international organizations and health care providers aware of the existence of inequalities in the access to biologic medications in different area of the world and of the importance of the availability of these medications to improve the outcome of children living in poorer countries. One of the main aims of the study is to promote the regular use of quantitative clinical measures and the incorporation of parent- and child-reported outcomes in routine clinical care. It is now widely agreed that the shift toward a clinical care based on quantitative measurements leads to a more rational monitoring of the disease course over time and the effectiveness of therapeutic interventions. Notably, the use of a standardized and uniform set of clinical instruments across international pediatric rheumatology centers is fundamental to ensure that the data regarding disease characteristics, outcome and treatment results are comparable. The ultimate aim of the EPOCA study is to contribute to improve the quality of care of children with JIA.

## Competing interests

The authors declare no conflict of interest.

## Author's contribution

AC, NR, GF, AM, and AR have made substantial contributions to study conception and design; AC, SL, GB, MG, SS, LV, GS, DT, and AZ gave substantial contribution to acquisition of data; AC, NR, GF, SL, GB, MG, SS, LV, GS, DT, AZ, AM, and AR have been involved in drafting the manuscript or revising it critically for important intellectual content, AC, NR, GF, SL, GB, MG, SS, LV, GS, DT, AZ, AM, and AR gave final approval of the version of the article to be published.
